# Aicardi syndrome in a 7-month-old girl with tonic seizures and skeletal defects: A case report

**DOI:** 10.1016/j.amsu.2021.102447

**Published:** 2021-05-28

**Authors:** Saeed Saado, Albaraa Bara, Yazane Abdallah

**Affiliations:** a Faculty of Medicine, Damascus University, Damascus, Syrian Arab Republic; b Radiologist at Damascus University Children's Hospital, Damascus, Syrian Arab Republic

**Keywords:** Case report, Aicardi, Seizures, Corpus callosum, Development

## Abstract

**Introduction:**

and importance: Aicardi syndrome (AS) is a rare genetic syndrome characterized by a triad of features: agenesis or hypogenesis of corpus callosum, chorioretinal lacunae, and infantile spasms, along with other neurodevelopmental, ocular, craniofacial, gastrointestinal, and musculoskeletal disorders. The precise etiology of AS is unknown, and establishing a diagnosis is challenging since it is an extremely rare syndrome and may mimic other congenital neurological defects.

**Case presentation:**

A 2-month-old girl was brought to our hospital, she developed multiple episodes of generalized spasticity with hyperflexion of upper and lower extremities on trunk (tonic seizure), with fast jerking movements of the eye, with signs of Psychomotor Development delay.

**Clinical discussion:**

Ophthalmic examination showed bilateral medial strabismus without nystagmus, Retinal examination showed bilateral small peripapillary well-circumscribed chorioretinal lacunae with hyperpigmented borders, thoracic spine x-ray (AP view) showed hemivertebrae and loss of height in the 7th and 8th thoracic vertebral body. Magnetic Resonance Imaging (MRI) revealed grey matter heterotopia, underdevelopment of the left operculum, hypogenesis of the corpus callosum, hypogenesis of inferior vermis, and multiple cysts with peripheral enhancing. Eventually, the diagnosis of AS is confirmed by the results of radiological imaging and ophthalmology exam.

**Conclusion:**

In this paper, we report a case of Aicardi syndrome diagnosed in a 7-month-old girl with frequent tonic seizures. We report this case to highlight that Aicardi syndrome should be considered in the differential diagnosis in cases of frequent tonic seizures with abnormal findings on retinal examination and characteristic findings on MRI.

## Highlights

• Aicardi syndrome (AS) is a rare syndrome characterized by a triad of features.
• AS triad is callosal hypogenesis, chorioretinal lacunae, and infantile spasms.
• Establishing the diagnosis of AS is challenging.
• AS may mimic other congenital neurological defects.

## 1 Introduction

Aicardi syndrome (AS) is an extremely rare genetic syndrome with multiple neurodevelopment disorders. It was first described in 1965 by Dr. Jean Aicardi as a triad of features: agenesis or hypogenesis of corpus callosum, chorioretinal lacunae, and infantile spasms [1].

The precise causative chromosomal abnormality responsible for AS is not exactly known, but it has been hypothesized that AS is a result of de novo mutation on the chromosome X, therefore, it primarily affects females with a few exceptions of males with Klinefelter syndrome (47, XXY) [2]. Drug resistant epilepsy is usually seen in severe AS cases, along with severe cognitive impairment and reduced life expectancy [3]. However, AS could manifest with wide variability of disease severity and multiples combinations of neurodevelopmental, ocular, craniofacial, gastrointestinal, and musculoskeletal clinical findings [4].

Establishing a diagnosis is challenging since AS is rare and may mimic Dandy-Walker syndrome, agenesis of the corpus callosum (ACC), Lennox- Gastaut syndrome (LGS), and more. However, the diagnosis is based on clinical manifestations along with radiological imaging and ophthalmology exam.

In this paper, we report a case of Aicardi syndrome diagnosed in a 7-month-old girl with frequent tonic seizures. The diagnosis was confirmed by ophthalmology exam and MRI. This work has been reported in line with the CARE criteria.

## 2 Case presentation

A girl was born at full term by vaginal delivery. At birth, she suffered a screaming delay for 5 minutes after delivery, with no cyanosis, and no oxygen or incubator needed. The girl has no surgical, family, or psychosocial history. At the age of 2 months, she developed multiple episodes of generalized spasticity with hyperflexion of upper and lower extremities on trunk (tonic seizure) with fast jerking movements of the eye, and no cyanosis, fever, or loss of consciousness. in this stage, the frequency of Seizures reached 7 episodes a day, with a duration of 10 minutes for each one. The baby was then put on 1mg\day of phenobarbital without remarkable improvement. After 1 week, the dose was raised to 2mg\day and seizures were down to 2 episodes a day, and continued on this situation for around 5 months.

At the age of 7 months, she was evaluated in Pediatric hospital in Damascus. Her weight = 6.5 kg, height = 65 cm, and head circumference = 39.5 cm, she had a broad flat nasal bridge with wide mouth, and signs of Psychomotor Development delay; difficulty in standing with support and holding head up, and can't roll from stomach to back and vice versa.

The clinical impression was pointing towards a congenital neurological defect. For further assessment, we did an ophthalmic exam, a thoracic spine x-ray, and an MRI.

Ophthalmic examination showed bilateral medial strabismus without nystagmus, with normal optokinetic responses and reactive pupils. Retinal examination showed bilateral small peripapillary well-circumscribed chorioretinal lacunae with hyperpigmented borders, the optic disc was normal thoracic spine x-ray (AP view) showed hemivertebrae and loss of height in the 7th and 8th thoracic vertebral body.

MRI revealed subependymal grey matter heterotopia with a nodular pattern (Fig. 1A), underdevelopment of the left operculum (Fig. 1B), hypogenesis of the inferior vermis (Fig. 1C), hypogenesis of the posterior part of corpus callosum (Fig. 2A), and multiple cysts with peripheral enhancing, two of them were interhemispheric, one in the posterior horn of left ventricle, and one in the posterior horn of right ventricle (Fig. 2B, Fig. 2CB&C).

**Fig. 1A:**
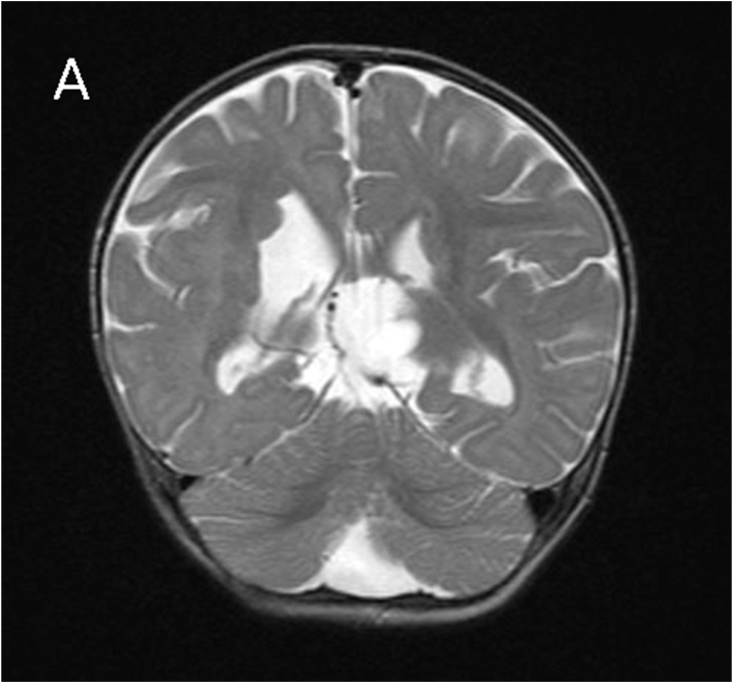
Magnetic resonance imaging (MRI) for our patient at the age of 7 months. A: coronal T2-weighted image showing subependymal grey matter heterotopia.

**Fig. 1B:**
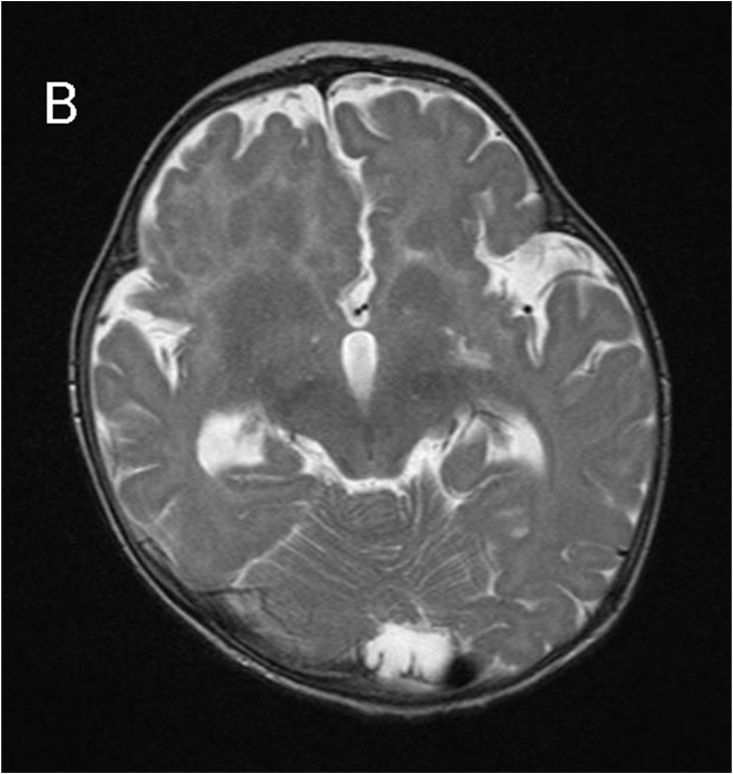
transverse T2-weighted image showing underdevelopment of the left operculum.

**Fig. 1C:**
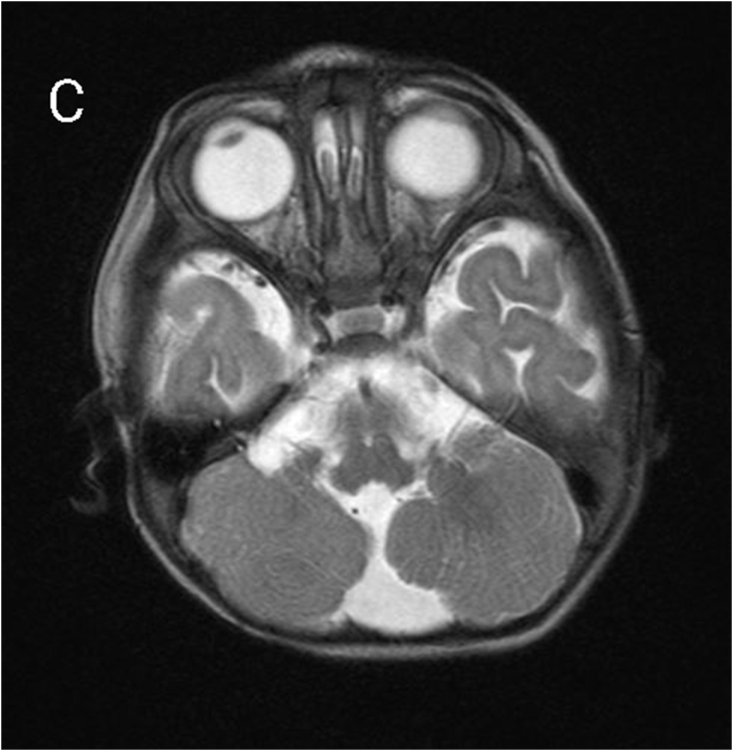
transverse T2-weighted image showing hypogenesis of the inferior vermis.

**Fig. 2A:**
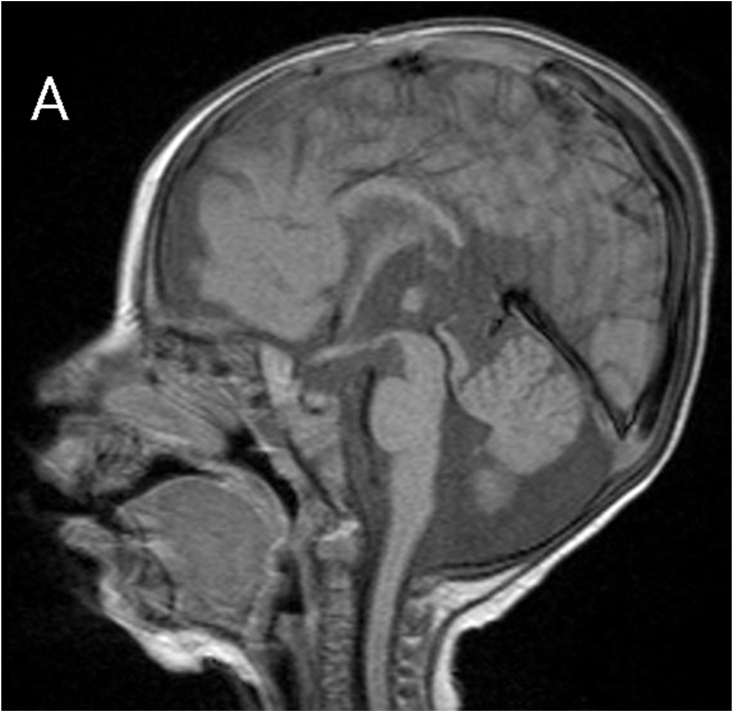
Magnetic resonance imaging (MRI) for our patient at the age of 7 months. A: sagittal T1-weighted image showing hypogenesis of corpus callosum.

**Fig. 2B:**
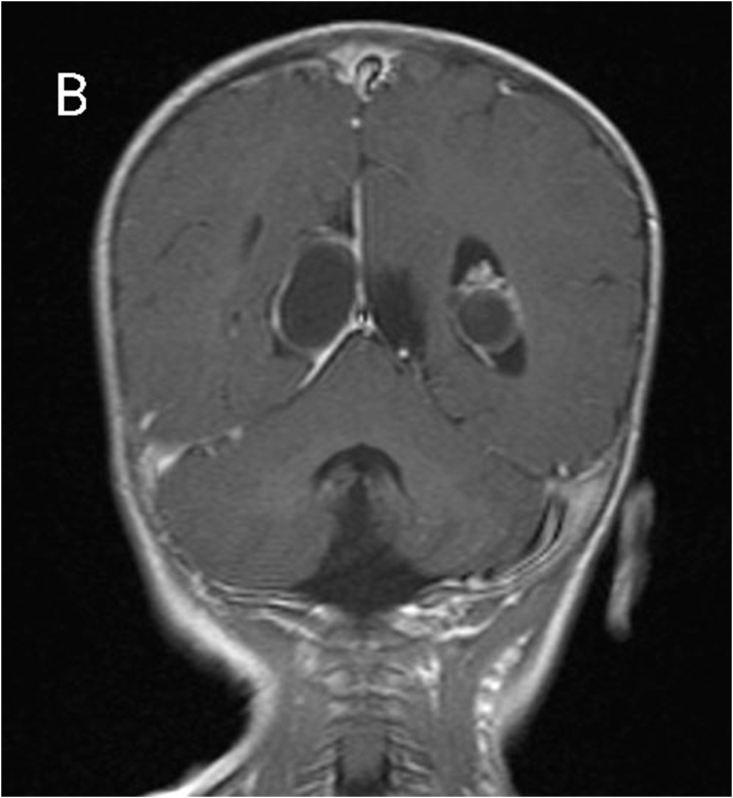
coronal T1-weighted image showing multiple cysts with peripheral enhancing.

**Fig. 2C:**
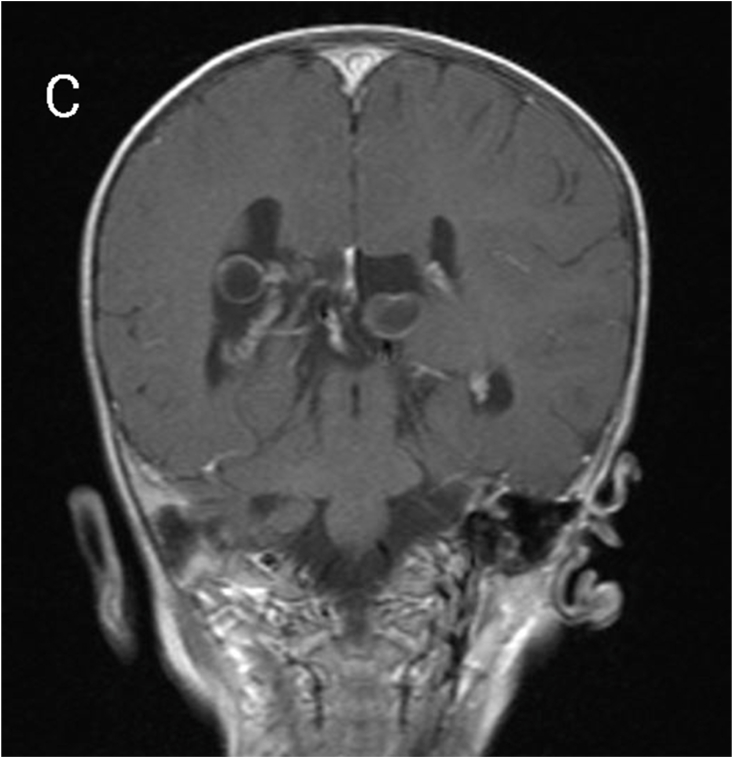
Coronal T1-weighted image showing multiple cysts with peripheral enhancing.

The presence of these characteristic findings on MRI, along with the chorioretinal lacunae and the frequent tonic seizures confirmed the diagnosis of Aicardi syndrome, and excluded the differentials (Dandy-Walker syndrome, agenesis of the corpus callosum, Lennox- Gastaut syndrome). As a follow-up, the seizures are being reassessed regularly, and psychomotor development is being monitored in her visits every 3 months.

## 3 Discussion

In 1946, Krause first described the ocular findings of Aicardi syndrome in an infant girl with seizures, developmental delay, and grey-white plaques in the retina bilaterally [5].

In 1965, Aicardi et al. documented the classic findings of this syndrome in a series of girls with infantile spasms, absence of the corpus callosum on pneumoencephalogram, and abnormal eye findings (microphthalmia, coloboma, and atrophic choroiditis) [1].

AS is characterized by three main features; agenesis or hypogenesis of the corpus callosum, seizures which begin in infancy (infantile seizures), and tend to progress to recurrent seizures (epilepsy) that can be difficult to treat, as the case we previously presented, and the last characteristic is chorioretinal lacunae, which are defects in light sensitive tissue at the back of the eye [6].

Most of AS patients develop normally until 3 months of age, and then, epileptic seizures and developmental delay start to emerge [7], however, in our case, the baby developed spasm-like epileptic seizures at the age of two months.

The corpus callosum normally develops from the anterior to posterior direction. In our case, hypogenesis was in posterior corpus callosum and commissure with a normal anterior section.

Callosal dysgenesis is typically associated with interhemispheric cysts and grey matter heterotopia [8], similar to our case, however.

Costovertebral malformations such as hemivertebrae, kyphoscoliosis, fusion of vertebrae, and absent ribs could be seen in AS, in our case we have seen hemivertebrae and loss of height in the 7th and 8th thoracic vertebral body.

The prognosis in AS cases varies with the severity of brain abnormalities and underlying symptoms, and life expectancy could be severely limited from several months to only a few years [9]. In our case, the baby developed epilepsy in a very young age, which has a bad prognosis, but she had a partial hypogenesis in the corpus callosum with small chorioretinal lacunae, which have a favorable prognosis.

## 4 Conclusion

We report this case to highlight that Aicardi syndrome should be considered in the differential diagnosis in cases of frequent tonic seizures with abnormal findings on retinal examination and characteristic findings on MRI.

## Ethical approval

None.

## Sources of funding

None.

## Author statement

SS: reviewed the literature, wrote the case presentation, and the discussion.

AB: reviewed the literature, wrote the abstract, and the introduction.

YA: checked the spelling and grammar, wrote the conclusion, designed the figures and provided the captions of the figures.

## Trial registry number

None.

## Informed consent

Written informed consent was obtained from the patient's mother for publication of this case report and accompanying images. A copy of the written consent is available for review by the Editor-in-Chief of this journal on request.

## Provenance and peer review

Not commissioned, externally peer-reviewed.

## Declaration of competing interest

All of the authors declared that they have no conflict of interest.
